# Differential Susceptibility to the Impact of the COVID-19 Pandemic on Working Memory, Empathy, and Perceived Stress: The Role of Cortisol and Resilience

**DOI:** 10.3390/brainsci11030348

**Published:** 2021-03-09

**Authors:** Shishir Baliyan, José Manuel Cimadevilla, Silvia de Vidania, Matías M. Pulopulos, Carmen Sandi, César Venero

**Affiliations:** 1 Department of Psychobiology, Universidad Nacional de Educación a Distancia (UNED), 28040 Madrid, Spain; 2 Department of Psychology, University of Almeria, 04120 Almeria, Spain; jcimadev@ual.es; 3 Health Research Center, University of Almeria, 04120 Almeria, Spain; 4 Molecular Neuropathology, Physiological and Pathological Processes, Centro de Biología Molecular Severo Ochoa, CSIC/UAM, 28049 Madrid, Spain; silvidba@gmail.com; 5 Department of Psychology and Sociology, University of Zaragoza, 50009 Zaragoza, Spain; matias.pulopulos@unizar.es; 6 Laboratory of Behavioral Genetics, Brain Mind Institute, Ecole Polytechnique Fédérale de Lausanne (EPFL), 1015 Lausanne, Switzerland; carmen.sandi@epfl.ch; 7 Instituto Mixto de Investigación-Escuela Nacional de Sanidad (IMIENS), 28029 Madrid, Spain

**Keywords:** social confinement, COVID-19, individual differences, stress, cortisol, working memory, perspective-taking, empathic concern, perceived stress, anxiety, depression, empathy

## Abstract

There are important individual differences in adaptation and reactivity to stressful challenges. Being subjected to strict social confinement is a distressful psychological experience leading to reduced emotional well-being, but it is not known how it can affect the cognitive and empathic tendencies of different individuals. Cortisol, a key glucocorticoid in humans, is a strong modulator of brain function, behavior, and cognition, and the diurnal cortisol rhythm has been postulated to interact with environmental stressors to predict stress adaptation. The present study investigates in 45 young adults (21.09 years old, SD = 6.42) whether pre-pandemic diurnal cortisol indices, overall diurnal cortisol secretion (AUCg) and cortisol awakening response (CAR) can predict individuals’ differential susceptibility to the impact of strict social confinement during the Coronavirus Disease 2019 (COVID-19) pandemic on working memory, empathy, and perceived stress. We observed that, following long-term home confinement, there was an increase in subjects’ perceived stress and cognitive empathy scores, as well as an improvement in visuospatial working memory. Moreover, during confinement, resilient coping moderated the relationship between perceived stress scores and pre-pandemic AUCg and CAR. In addition, in mediation models, we observed a direct effect of AUCg and an indirect effect of both CAR and AUCg, on change in perceived self-efficacy. These effects were parallelly mediated by the increase in working memory span and cognitive empathy. In summary, our findings reveal the role of the diurnal pattern of cortisol in predicting the emotional impact of the COVID-19 pandemic, highlighting a potential biomarker for the identification of at-risk groups following public health crises.

## 1. Introduction

The ongoing Coronavirus Disease 2019 (COVID-19) pandemic can be considered a long-term psychosocial stressor. In only a few months from its start, there were already reports indicating its negative impact on mental health, including increased rates of anxiety, depression, and stress perception [1,2]. These studies add to the extensive literature about the behavioral and cognitive effects of stress exposure [3,4,5,6]. Specifically, a growing number of studies have shown that stress can impact social behaviors (e.g., prosocial behavior [7]; empathy [8,9]) and cognition, especially for executive functions (e.g., decision-making [10] and working memory [11,12]). However, the vast majority of research has been carried out under laboratory settings, and there is little evidence about cognitive aspects of the pandemic’s impact on these behavioral and cognitive functions or the relationship between the cognitive and emotional conditions generated.

There are important individual differences in the effects of chronic stress on the brain, behavior, and cognition [13,14,15]. The pattern of glucocorticoid responsiveness to stress [16,17,18,19] and the diurnal rhythm of cortisol [20,21,22] are gaining attention for their utility as biomarkers not only of the stress response but also of cognitive state and emotional well-being.

Indeed, cortisol is the main adrenal glucocorticoid hormone and final product of the hypothalamic–pituitary–adrenal (HPA) axis, a key neuroendocrine system of the stress response [23]. Cortisol shows a strong circadian rhythm, ranging from higher concentrations shortly after awakening (i.e., cortisol awakening response, or CAR) to lower levels in the evening, following a steady decay during daytime [24,25]. Regardless of circadian variations, the total diurnal cortisol release is estimated as the area under the curve with respect to ground (AUCg) [26]. Both CAR and AUCg are widely used indices of diurnal cortisol functioning, although they relate to distinct biological processes [26,27]. The CAR, an acute increase in cortisol levels that peaks 30–45 min after awakening, is typically a stable readout [25] and a useful index of HPA axis functioning [20,27]. Thus, perceived stress has been linked to greater CAR in both chronically stressed individuals [28,29] and healthy young adults [30]. In addition, CAR has been shown to predict major depressive disorder [31,32] and heart rate variability [33], the latter a marker of mental health resilience [34]. Importantly, an interaction between AUCg and experiences of negative life events has been found to predict the onset of depression [35]. Moreover, a flattening of the diurnal cortisol rhythm has been reported to predict cancer survival [36,37], while waking cortisol levels can predict emotional responses to potentially traumatic events [38], post-traumatic stress disorder (PTSD) [39], and even socioemotional adjustment [40]. However, the possibility that individual pre-pandemic (baseline) diurnal cortisol profiles may predict posterior self-perceived stress after being exposed to a stressful situation (e.g., pandemic) has remained largely unexplored.

Cognitive capacities are related to the individual’s emotional aspects of personality [41], as in empathy, a process consisting of both emotional (being moved by another’s emotions) and cognitive processes (knowing another’s emotions) [42]. Cognitive empathic processing is associated with prefrontal cortex activity [43], and it is related to working memory and cognitive flexibility [44]. Manipulation of either cognitive capacity or emotional state coincides with concurrent changes in the other. Specifically, Allott et al. (2015) [45] showed an inverse relationship between working memory and perceived stress. Similarly, an individual’s cognitive abilities are known to buffer them against potential negative effects of stress [46], while the problem-solving ability is inversely related to posteriorly self-perceived stress [47]. However, as far as we know, no previous studies have explored whether, in a long-term stressful event, diurnal cortisol indices can be used as prognostic biomarkers for changes in cognitive function and how these changes relate stress perception in healthy young adults.

Hence, the main objective in this study was to determine if diurnal cortisol profiles of individuals before the COVID-19 pandemic could help to predict the impact of the strict social confinement during the first wave of the pandemic on (i) perceived stress (as reported using the Perceived Stress Scale; PSS); (ii) empathy (as measured by the two self-reported subscales of the interpersonal reactivity index (IRI) measuring cognitive empathy (Perspective-Taking) and emotional empathy (Empathic Concern) [48]; (iii) attention (as measured by the change-location task), and (iv) working memory (as measured by the Corsi block-tapping test).

**Figure 1:**
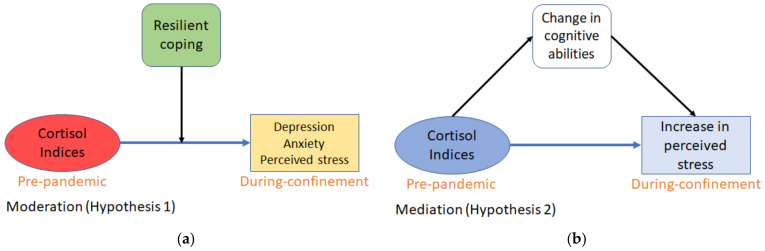
Conceptual models for the theoretical framework of the hypotheses: (**a**) Coping abilities of subjects will moderate the influence of pre-pandemic cortisol indices on self-reported depressive-like symptoms, anxiety, and perceived stress during pandemic + confinement; (**b**) Change in cognitive capacities during pandemic + confinement will mediate the change in perceived stress as caused by pre-pandemic cortisol indices.

The present study examined the relation of pre-pandemic diurnal cortisol indices (CAR and AUCg) and the psychological (perceived anxiety, stress, and depression-like symptoms) impact of the strict social confinement during the first wave of the COVID-19 pandemic (until May 2020, see 2.2 Procedure for details). Spain registered its first COVID-19 case on 27 February 2020, and on 14 March, the government declared a countrywide state of alarm comprising strict confinement (or lockdown) lasting ~50 days. Thus, given the predictive capacity of cortisol for the development of depression/anxiety and research showing how these emotions correlate with perceived stress, we hypothesized that (Hypothesis (1)) pre-pandemic diurnal cortisol indices would predict not only depressive-like symptoms and anxiety, but also perceived stress during the confinement, and that this relationship would be moderated by resilient coping (Figure 1a). Additionally, given that cognitive abilities can buffer the impact of stress on emotion [46] and that CAR has been related to spatial working memory [49,50,51] (but also see [52]), we hypothesized that (Hypothesis (2)) change in cognitive abilities following confinement would mediate the relationship between pre-pandemic cortisol indices and the increase in perceived stress during confinement, such that improving cognition would buffer against worsening perceived stress (Figure 1b).

## 2. Materials and Methods

### 2.1. Participants

Seventy-nine undergraduate psychology course students (78% females; mean age 20.67; SD 5.11; range 18–52) were recruited to participate in a study investigating empathy and spatial working memory. During the confinement session, forty-five of these subjects, with an age range of 18–52, agreed to participate in this second assessment. Study sample characteristics and pre-pandemic (baseline) values for cortisol indices, anxiety, depression, and resilient coping are listed in Table 1. The students received course credit for participating in this study. Data during home confinement were collected at the very end of the strict confinement, at a time when the confinement measurements were relaxed less than 5 days after the average subject response date. No differences in the variables investigated in this study were observed between subjects who did not participate in the second assessment and the final sample included in the current investigation (Table S2, Supplementary Materials).

### 2.2. Procedure and Study Timeline

**Figure 2:**
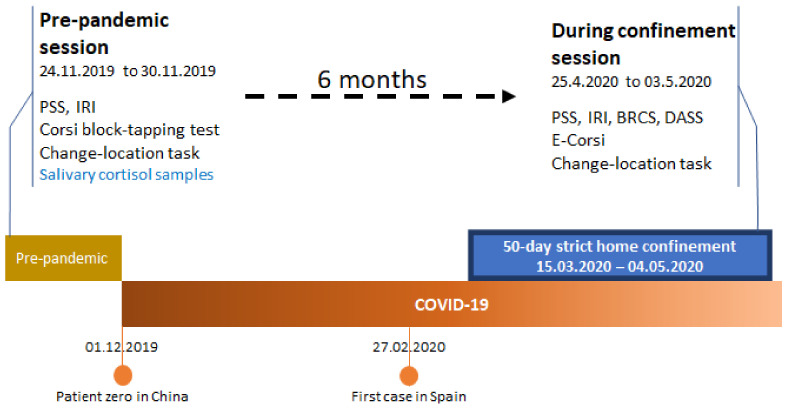
Study-related procedures in a timeline.

This study was composed of two sessions, pre-pandemic and during-confinement (Figure 2). The pre-pandemic assessment was conducted in-person and, following the collection of demographic data, subjects carried out the cognitive tests and responded to questionnaire measures. All questionnaires were responded to by the participants alone in a room. Thus, perceived stress, empathic proclivity, working memory capabilities, and five same-day saliva samples had been collected just before the beginning of the pandemic (24.11.2019 to 30.11.2019). All students who participated in the pre-pandemic study received an e-mail 6 months later, inviting participation in a study concerning the COVID-19 pandemic. Subsequently, we asked these participants to answer relevant questionnaires and undergo cognitive testing towards the end of the first 50-day government decreed strict lockdown (25.4.2020 to 3.05.2020) (Figure 2). Subjects completed the during-confinement session at home.

### 2.3. Saliva Sample Collection

At the end of the pre-pandemic session, all subjects were provided saliva collection tubes with verbal as well and written instruction about how to collect the 5 samples. Subjects collected their samples on awakening, +30 min, +45 min, 7 h after waking, and right before going to sleep and were asked to do this on a typical day. Subjects were told not to eat, smoke, take any stimulants (such as coffee, caffeinated drinks, or tea) or brush their teeth at least 1 h before sample collection. CAR was calculated using the area under the curve with respect to the increase, as suggested by Pruessner and colleagues [53].

### 2.4. Cognitive Tests

#### 2.4.1. Corsi Block-Tapping Test

Participants were instructed about how to carry out the Corsi block-tapping test in the forward and backward order. The equipment used consisted of a wooden board (23 × 28 cm) upon which nine cubes were arranged in an irregular pattern [54]. For the Corsi-forward procedure, the evaluator tapped the cubes in sequences of increasing length (from 2 to 9 cubes), and subjects were asked to tap them in the same serial order following the demonstration. Two trials with different orders (of equal length) were performed for each sequence, and in case of a correct response, the number of cubes tapped increased by 1 in the following sequence. The Corsi-backward procedure consisted of asking the subject to tap the cubes in the order inverse to that demonstrated. The result was scored for Corsi-forward, Corsi-backward, and the total, i.e., the sum of sequences tapped correctly in either pattern. Thus, the range of scores obtainable was 0–14 on both Corsi-forward and backward and 0–34 on Total Corsi score. Testing was stopped when the subject was unable to advance to the next series after two failed attempts.

#### 2.4.2. Change-Location Task

Scope and control of attention were measured by employing the change-location visual array task [55]. This task is a measure of the maintenance capacity of working memory [56], and it implicates neither distractions nor higher-order complex processing. Such tests with 1–3 circles tend to reveal very high accuracy across most subjects. However, 4 circles cause accuracy to decline, implying that this is where focal attention of subjects becomes challenged. For this change-location task, a fixation point was shown for 1000 ms, followed by the display of four differently colored circles for 150 ms. A black screen was presented next, for 900 ms, followed again by four circles, with only one being of a different color than earlier. Subjects were instructed to choose which circle had changed in color. Participants received verbal instructions followed by 12 practice trials followed by 64 test trials divided into 2 equal blocks. This task was carried out using a personal computer and in the absence of the instructor to avoid possible distractions. The task duration was about nine minutes. The number of correct responses and errors was registered, and the K-index ((proportion correct × number of circles) – 1) was indicative of performance at the task. The range of K-index scores obtainable was 0–4.

#### 2.4.3. Electronic Corsi Block-Tapping Test (e-Corsi)

The e-Corsi was similar to the traditional Corsi block tapping test. The forward and backward trials were presented as sequences of squares highlighted in a specific order. At the sequence end, the participants were instructed to reproduce the sequence by clicking the squares in the order they had flashed. The range and calculation of Corsi-forward, backward and total Corsi scores were as described for the traditional Corsi block-tapping task.

Both the above tasks were carried out via online methods (e-Corsi was employed in place of traditional Corsi, refer to discussion for information about the absence of repetition and digitization effects) in the amid-confinement phase of the study.

### 2.5. Questionnaire Measures

#### 2.5.1. Perceived Stress Scale (PSS)

Participants responded to the Spanish version of the self-reported perceived stress scale [57] at home during the pre-pandemic as well as confinement stages of the study. The scale requested subjects to report the frequency (in the last 30 days) with which they had certain thoughts and feelings using 14 items describing daily life situations. Subjects replied on a 5-point Likert-type scale, and the total score was obtained by reversing the inverse items and adding the scores of the 14 items, higher totals signify higher perceived stress. The PSS consisted of positively-worded (the inverse items) and negatively-worded items, which represent the two constructs measured, perceived self-efficacy (also known as perceived coping) and perceived helplessness (also known as perceived distress) [58]. Perceived self-efficacy can be defined as the subjective interpretation of how capable one is in dealing with prospective situations [59].

#### 2.5.2. Interpersonal Reactivity Index (IRI)

The Spanish version of IRI [60] was used to measure cognitive and emotional empathy via the sub-scales, Empathic Concern (EC) and Perspective-Taking (PT), respectively [48]. Participants completed both subscales during the pre-pandemic and the confinement. Each sub-scale consisted of 7 items with subjects instructed to reply on a 5-point Likert-type scale about how adequately each item described them. Each subscale had a possible score range of 0–28.

#### 2.5.3. Brief Resilient Coping Scale (BRCS)

Resilience was measured using the validated Spanish adaptation of the Brief Resilient Coping Scale, BRCS [61]. This 4-item scale was used only during the during-confinement session. The possible score range was from 4–20, where a higher score meant greater resilient coping. The possible score range was from 4–20, where a higher score meant greater resilient coping.

#### 2.5.4. Depression Anxiety and Stress Scales (DASS)

Depression-like symptoms and anxiety levels were measured using the Spanish version of the depression anxiety and stress scale [62]. It consisted of 14 questions, 7 items for either scale. To lessen the burden of the participants, we did not administer the stress scale since we were already using the PSS. This scale was used only during the during-confinement session. Participants were instructed to use a 4-point Likert-type scale to report how accurately the situations described in the items reflected their feelings during the last month (instead of just the last week; changed to get data reflecting the effects of the pandemic and confinement in general and not only the effects of the latter part of the confinement). Both subscales had a possible score range of 0–21.

### 2.6. Data Management and Statistical Analyses

For cortisol levels, we calculated two indices using the cortisol samples collected on awakening, +30 min, +45 min, 7 h after waking and right before going to sleep: (i) the CAR (i.e., the dynamic of the cortisol increase after awakening) was computed as the area under the curve with respect to the increase using the salivary samples collected at awakening, and 30 and 45 min after awakening, and; (ii) the area under the curve with respect to the ground using all the saliva samples collected during the day (AUCg) (see [53] for the formulas).

Pre-pandemic mean scores were compared with during-confinement values to evaluate the effects of the pandemic and confinement on perceived stress, empathy, working memory, and attention. Given that we had access to both pre-pandemic and during confinement data of the same subjects, we employed paired samples *t*-tests or Wilcoxon’s test where applicable. The change in scores was calculated by subtracting the pre-pandemic values from the during-confinement condition enabling us to quantify the magnitude of the changes and use these as variables. The significance level was set at *p* ≤ 0.05, two-tailed, for all analyses.

To test our proposed moderation and mediation models, we employed a regression-based path analysis using the PROCESS plugin (version 3.5), a macro for estimating and probing interactions [63], for SPSS. We estimated model 1 for moderation (Hypothesis 1) and model 4 for parallel mediation (Hypothesis 2) in PROCESS employing 5000 bootstrap samples and 95% bias-corrected bootstrap confidence intervals (CI).

In moderation models, we investigated whether the relation of cortisol indices (i.e., CAR and AUCg) to depression-like symptoms and anxiety (i.e., DASS) and perceived stress (i.e., PSS) were moderated by resilient coping (i.e., BRCS). The use of a moderating variable widens the scope of the predictive value of cortisol indices on the emotional condition state during confinement.

Although cortisol indices could predict absolute scores of perceived stress during confinement, attention to the pre-pandemic values was needed to study the dynamic changes induced by the pandemic. Thus, to investigate whether cortisol indices were able to predict the impact of the COVID-19 pandemic, we decided to use the change in perceived stress from the pre-pandemic condition to the confined condition as the dependent variable in a mediation analysis. As previously indicated in the introduction, individuals’ cognitive abilities, including working memory, can ameliorate the impact of stress on emotion [46], and CAR has been related to spatial working memory [49,50,51]. Therefore, using mediation models, we could investigate if cortisol levels (i.e., CAR and AUCg) were related to the changes in perceived stress (i.e., PSS) via changes in executive function performance (i.e., Corsi-scores) and cognitive empathy (i.e., IRI-PT).

No univariate or multivariate outliers were detected in the variables used in the analyses. Statistical analyses were performed using the statistical package for social sciences (SPSS) version 25.0 (IBM, Armonk, NY, USA).

## 3. Results

### 3.1. Changes Across Pre-Pandemic and During-Confinement Scores

**Figure 3:**
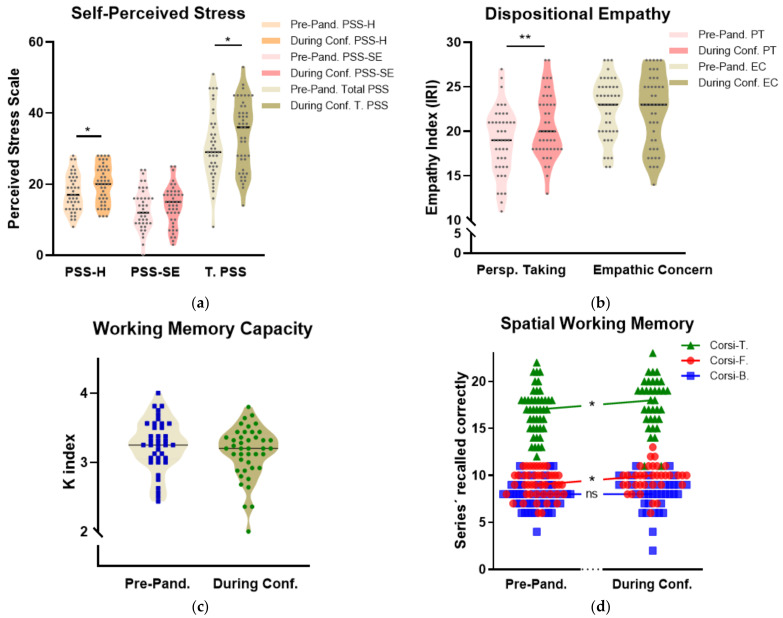
Pre-confinement and during-confinement scores: (**a**) Violin graphs (with median) showing an increase in perceived helplessness (PSS-H) and total perceived stress (T. PSS) (**b**) An increase in the perspective-taking (PT) aspect of dispositional empathy (**c**) Scores on the change-location task and (**d**) An increase in the number of block-sequences correctly recalled during Corsi-Forward and Corsi-Total. Note: Conf. = Confinement; PSS = Perceived Stress Scale; PSS-SE = Perceived (lack-of) Self-Efficacy; PSS-H = Perceived Helplessness; IRI = Interpersonal Reactivity Index; PT = Perspective-Taking; EC: Empathic Concern; Corsi-T = Total Corsi Score; Corsi-F = Corsi-Forward Score; Corsi-B = Corsi-Backward Score. * *p* < 0.05; ** *p* < 0.01.

To explore the neuropsychological impact of COVID-19 in our cohort, a paired *t*-test was used to determine whether the mean pre-pandemic values of the selected variables were different to the during-confinement scores (Table 2 and Figure 3). We analyzed variables of perceived stress (perceived helplessness, perceived self-efficacy, and total perceived stress), empathy (perspective-taking; cognitive empathy and empathic concern; emotional empathy), attention capacity (change location task), and spatial working memory (Corsi block-tapping test forward, backward, and total). Paired samples tests indicated a significant increase in self-perceived helplessness and total perceived stress (t(40) = −3.707, *p* = 0.004 and z = −2.563, *p* = 0.01, respectively), but there were no significant changes in perceived self-efficacy (z = −1.861, *p* = 0.06) (Figure 3a). There was a significant increase in perspective-taking (t(44) = −3.431, *p* < 0.01; Table 2), whereas there was no change observed in Empathic Concern (z = −0.515, *p* = 0.61; Table 2) (Figure 3b). No significant changes were observed in attention (i.e., change location task) (z = −1.414, *p* = 0.157; Table 2) (Figure 3c). Finally, there were significant improvements in during-confinement Corsi-forward (t(34) = −2.714, *p* = 0.10, Table 2) and total Corsi scores (t(34) = −2.675, *p* = 0.011; Table 2) when compared to their pre-pandemic values. Corsi-backward scores did not show any significant change between during-confinement and pre-pandemic conditions (t(34) = −0.719, *p* = 0.477; Table 2) (Figure 3d).

### 3.2. Moderation Analyses

Subsequently, we wanted to investigate whether pre-pandemic cortisol indices (AUCg, CAR) predict depressive-like symptoms, anxiety levels, and total perceived stress state reported during confinement. Using moderation models, we investigated Hypothesis 1 (Figure 1a); if pre-pandemic cortisol indices (AUCg, CAR) were related to during-quarantine absolute scores of total perceived stress, depression-symptoms, and anxiety, and whether these relationships were moderated by resilient coping capacity (i.e., BRCS score) (six independent moderation analyses were performed). Figures are presented for AUCg and CAR models in relation to total during-confinement perceived stress (moderated by resilient coping capacity). Other significant models’ (i.e., AUCg to DASS-depression and DASS-anxiety) tables and graphs are available in the Supplementary Data File (Table S1 and Figure S1).

**Figure 4:**
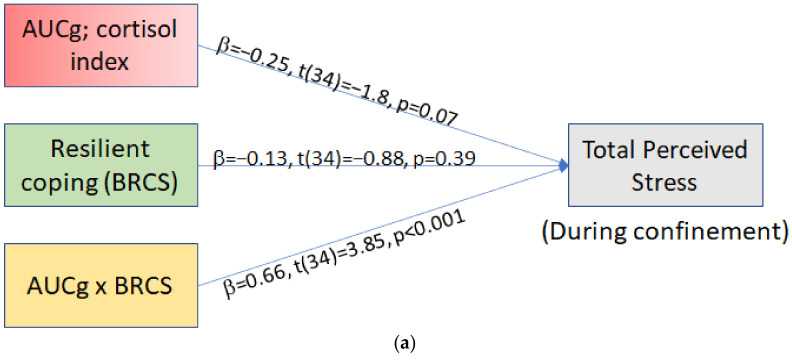

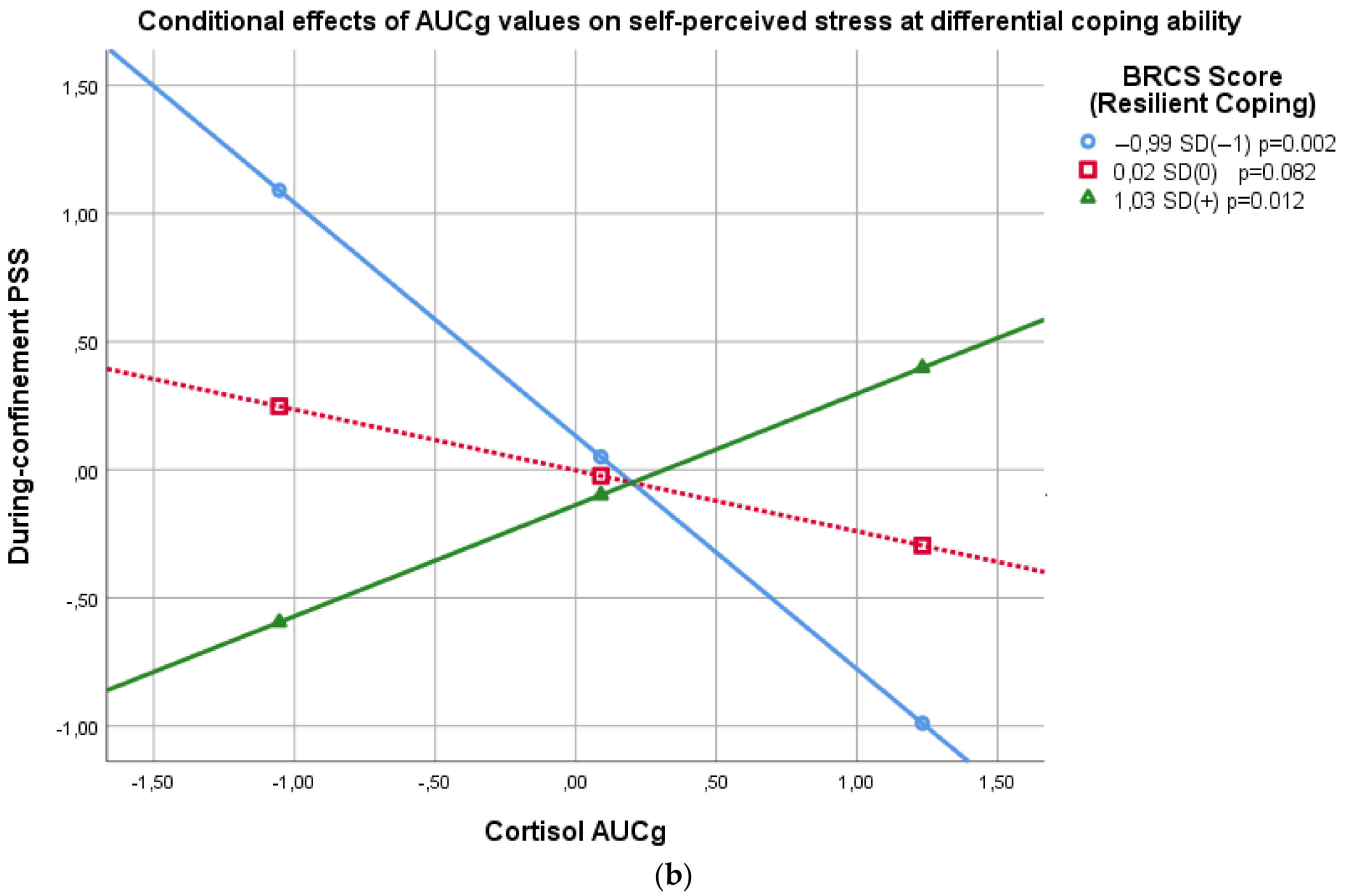
(**a**) Simple moderation analysis statistical model with standardized regression coefficients. (**b**) Simple slopes (conditional effects) representing the association between Resilient Coping and pre-pandemic daytime cortisol total diurnal cortisol release (AUCg), predicting confinement total perceived stress. BRCS = Brief Resilient Coping Score; AUCg = Total diurnal cortisol release; PSS = Perceived Stress Score.

AUCg. The overall model was significant (*F*(3,34) =7.87, *p* = 0.0004), showing that 41% of the variance in during-confinement perceived stress was predicted by AUCg, resilience score, and their interaction (Figure 4a). The interaction significantly predicted the total during-confinement perceived stress state (*p* = 0.0005) and was responsible for 26% of the variance in perceived stress after the confinement.

Simple slopes (at mean and ± 1 SD BRCS score) are presented in Figure 4b. To give the reader the opportunity to interpret raw scores, we describe below the results with the standardized values (b) as well as the unstandardized values (b). Graphs of conditional effects with the unstandardized scores are available in the Supplementary Materials (Figure S5). The Johnson–Neyman significance regions analysis revealed that when resilient coping was less than a BRCS score of 13.72 (b = −0.05), AUCg and during-confinement PSS showed a significant negative relationship (b = −2.10, b = −0.29, *p* = 0.05). As resilience decreased, the relationship between AUCg and PSS became more inverted (b = −13.80, b = −1.88, *p* < 0.01). Interestingly, when BRCS score was more than 16.83 (b = 0.80), AUCg and during-confinement PSS were again significantly related, but positively (b = 2.11, b = 0.29, *p* = 0.05). At the highest resilience scores (BRCS = 20; b = 1.67), AUCg and during-confinement PSS showed a significant and positive relationship (b = 6.35, b = 0.86 *p* < 0.01).

Significant moderation models were identified in the association between AUCg, depressive-like symptoms score, and anxiety (with resilient coping as moderator; figures and data in Supplementary Materials Table S1, Figures S1–S3).

**Figure 5:**
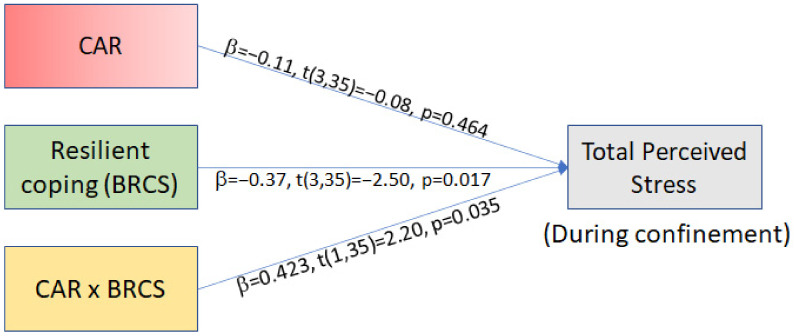
Simple moderation analysis statistical model with standardized regression coefficients. BRCS = Brief Resilient Coping Score, CAR = Cortisol Awakening Response.

CAR. The overall model was significant (*F*(3,35) =4.10, *p* = 0.0136), showing that 26% of the variance in during-confinement perceived stress was predicted by CAR, resilience score, and their interaction (Figure 5). The interaction significantly predicted total perceived stress scores during confinement (*p* = 0.035) and was responsible for 10% of the variance in perceived stress during the confinement. To give the reader the opportunity to interpret raw scores, we describe below the results with the standardized values (b) as well as the unstandardized values (b). Graphs of conditional effects with the unstandardized scores are available in the Supplementary Materials (Figure S5a). The Johnson–Neyman significance regions revealed that only when resilient coping was less than 8.25 (b = −1.55), CAR and during-confinement PSS were significantly related (b = −70.62, b = −0.77 *p* = 0.05). As resilience decreased, the relationship of CAR and PSS became more inverted with effect at lowest resilience (BRCS score 5; b = −2.45), (b = −105.6, b = −1.15 *p* = 0.041). CAR did not have any significant moderation models in relation to DASS scale score of Depression or Anxiety (Supplementary Materials Table S1).

Given the known impact of sex and age on cortisol [64,65], we also analyzed the moderation models with age and sex as covariables. All moderation models maintained similar results when run while controlling for age and sex (see Supplementary Materials; Table S3).

Moderation models were also run to explore how the two constructs of the PSS (i.e., perceived self-efficacy and perceived helplessness), measured by the two subscales, were related to cortisol and whether resilient coping moderates these relationships. Results revealed that the models were principally driven by perceived self-efficacy scores. Similar results were observed for CAR, where up to 43% of perceived self-efficacy was predicted by CAR, resilient coping, and their interaction against 26% for total PSS (see Supplementary Materials; Table S1, Figures S2 and S3). These results indicated that high perceived stress scores during confinement were observed in those individuals with low cortisol indices and low resilient capacity as well as in those with high cortisol indices and high resilient capacity. Overall, our moderation model indicates that resilient coping capacity seems to be a crucial factor to unravel the complex association between baseline diurnal cortisol indices and perceived stress state during a stressful situation.

### 3.3. Mediation Analyses

We investigated whether cortisol indices could predict the psychological stress impact of the COVID-19 pandemic and whether cognitive abilities (working memory and empathy) would mediate this relationship. In mediation analyses, we used the cortisol index as the predictor, the change in cognitive abilities as the mediators, and the change in perceived stress from the pre-pandemic condition to the confined condition as the dependent variable.

Hypothesis 2 (Figure 1b) was explored using mediation models. Specifically, we investigated whether CAR and AUCg were related to changes in perceived stress from the pre-pandemic condition to the confined condition via their relationship with change in performance on the Corsi block tapping task, a measure of executive function, and change in perspective-taking, a measure of cognitive empathy. We observed that changes in positive perceived stress (perceived self-efficacy) were predicted by the pre-pandemic cortisol indices via changes in both Corsi-forward and perspective-taking scores. Neither of the cortisol indices related to changes in total perceived stress scores via changes in total Corsi score.

**Figure 6:**
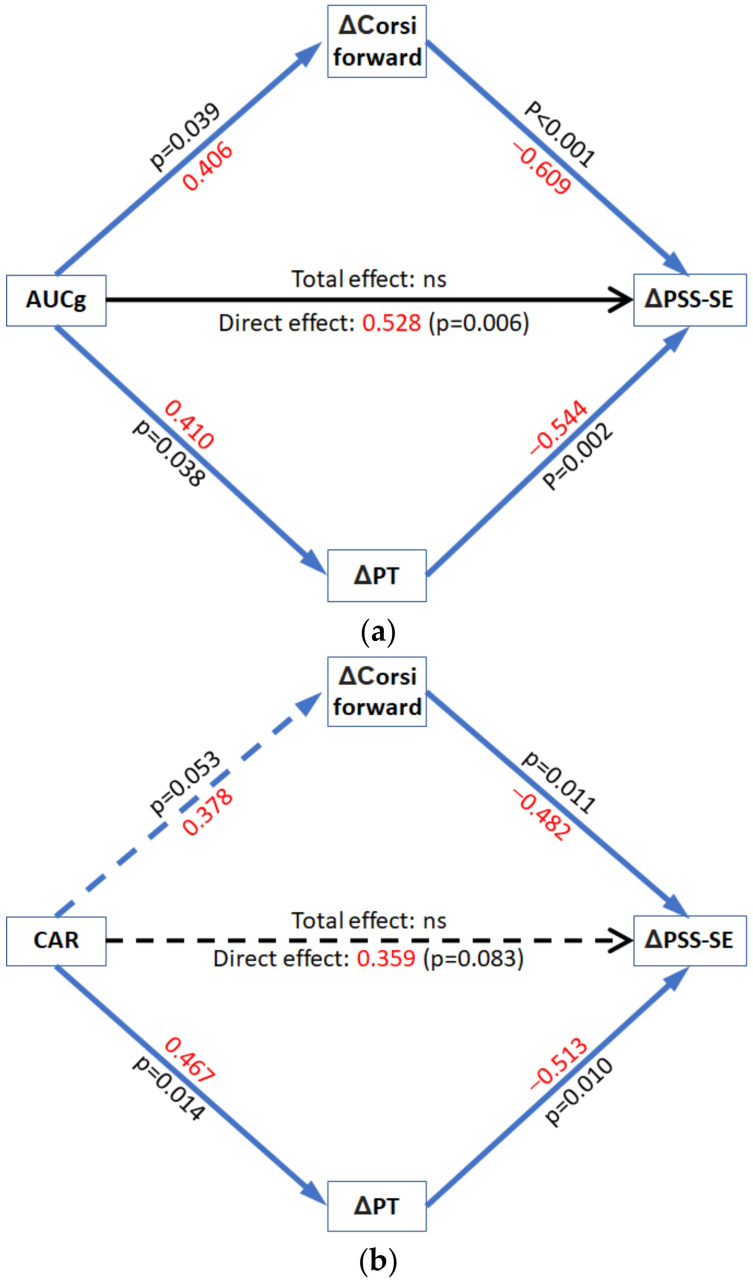
Mediation models (**a**) AUCg cortisol predicting change in perceived self-efficacy; (**b**) Cortisol awakening response (CAR) predicting change in perceived self-efficacy. PT = Perspective-Taking; PSS-SE = Perceived stress scale Self-Efficacy; AUCg = Total diurnal cortisol release; CAR = Cortisol Awakening Response; ns = non-significant. Standardized effect sizes in red.

AUCg. Figure 6a. We observed that higher AUCg was associated with greater increases in scores on Corsi-forward (*p* = 0.04) and perspective-taking (*p* = 0.04). In turn, greater positive change in performance on the Corsi-forward (*p* < 0.01) and greater positive change in perspective-taking (*p* < 0.01) were related to lower worsening in the self-reported perceived self-efficacy. Importantly, the mediation analyses indicated a significant indirect effect (ES = −0.470; 95% CI = [−0.710, −0.266]) of AUCg on changes in perceived self-efficacy via changes in Corsi-forward (ES = −0.247; 95% CI = [−0.480, −0.061]) and perspective-taking (ES = −0.223; 95% CI = [−0.439, −0.056]). These results indicated that a larger AUCg was associated with higher improvement in perspective-taking and spatial working memory following the confinement. These improvements, in turn, were related to a lower worsening of perceived self-efficacy following confinement. The overall model explains up to 54% of the variance seen in the change in self-reported perceived self-efficacy (F(3,22) =8.47, *p* = <0.01). Critically, the direct effects of AUCg on during-confinement change in perceived self-efficacy were also statistically significant (ES = 0.528, 95% CI = [0.623, 3.264], *p* < 0.01), and higher AUCg levels predicted higher change in perceived stress. The suppressing effect of the direct and indirect effects on each other led to a statistically non-significant total effects model (F(1,24) =0.0804, *p* = 0.78, r^2^ = 0.003). Thus, overall, AUCg did not directly predict a change in perceived self-efficacy following the COVID-19 confinement. There was no predictor/correlational relationship between the two mediators, and no interaction effects (between AUCg and either of the mediators) were observed.

CAR. Figure 6b. We observed that the CAR had a trend towards being positively associated with changes in scores on Corsi-forward (*p* = 0.053) and significantly predicted changes in perspective-taking (*p* = 0.01). Again, greater positive change in performance on the Corsi-forward (*p* = 0.01) and greater positive change in perspective-taking (*p* = 0.01) were related to a lower decrease in the self-reported perceived self-efficacy. Importantly, the mediation analyses indicated an indirect effect of CAR on changes in perceived self-efficacy via changes in Corsi-forward (ES = −0.181; 95% CI = [−0.373, −0.060]) and perspective-taking (ES = −0.239; 95% CI = [−0.447, −0.062]), giving a total indirect effect of (ES = −0.420; 95% CI = [−0.674, −0.193]). According to these results, a larger CAR was associated with higher improvement in perspective-taking and spatial working memory during confinement. These improvements, in turn, were related to a lower worsening of perceived self-efficacy. The overall model explained up to 39% of the variance seen in the change in self-reported perceived self-efficacy (F(3,23) =4.81, *p* = <0.01). The direct effect of CAR on during-confinement change in perceived self-efficacy was not statistically significant (ES = 0.359, 95% CI = [−1.99, 29.93], *p* = 0.08) and, although not significant, it had a trend toward an effect contrary to that which CAR had on PSS via a change in PT and Corsi-forward. The suppressing effect of the direct and indirect effects on each other led to a statistically non-significant total effects model (F(1,25) =4.815, *p* = 0.760, r^2^ = 0.003). Thus, overall, CAR did not directly predict change in perceived self-efficacy amid COVID−19 confinement. No relationship between the two mediators and no interaction effect (between CAR and either of the mediators) were observed.

The number of variates and covariates that can be included in the statistical models is restricted by the constrained sample size. Nevertheless, when controlling for age and sex, the mediation models of both CAR and AUCg lost the indirect effect via a change in Corsi-forward score, while the indirect effect via a change in perspective-taking and the direct effect of AUCg on change in perceived-self efficacy were still significant (see Supplementary Materials; Figure S5). Overall, our mediation model indicates that working memory span and cognitive empathy are two cognitive abilities that exert a key protective effect on individuals’ emotional response to the COVID-19 pandemic.

## 4. Discussion

Here, we revealed that individual cortisol profiles predict how long-lasting stressful circumstances impact perspective-taking, working memory and, eventually, perceived self-efficacy in dealing with prospective situations. Thus, our study goes beyond previous work indicating that the causal effects of stress on depression and anxiety [66,67] (for review see [68]) are moderated by resilience and coping style [69,70,71], by showing the predictive capacity of basal diurnal cortisol for the development of stress-related psychopathologies.

First, we explored the relationship between cortisol, resilient coping, and measures of depression, anxiety, and perceived stress. Resilient coping incorporates cognitive and behavioral strategies, such as active problem solving towards adverse and stressful circumstances [72]. Thus, we expected resilient coping to moderate the direction and intensity of cortisol’s prediction of mental health changes. We focused on CAR and AUCg as the principal cortisol indices, given their importance as risk factors predicting stress-related disorders, such as depression and post-traumatic stress disorder [35,38,39]. Additionally, treatment for depression and PTSD has been shown to correlate with normalization of cortisol profiles [73,74] and a decrease in perceived stress [75]. Our moderation models support Hypothesis 1 (Figure 1a), stating that pre-pandemic total diurnal cortisol secretion (i.e., AUCg) was associated with depressive-like symptoms, anxiety levels, and total perceived stress state reported during confinement. This relationship was moderated by resilient coping capacity in a manner that high coping corresponded to a positive relationship between the predictor AUCg and worsening of mental health. It is worth noting that low resilience scores, however, invert the relationship such that at low AUCg levels, subjects reported having high depressive-like symptoms/anxiety/perceived stress during home confinement. These observations could suggest that an inverted-U shape relationship between cortisol indices and distinct mental health out-comes (i.e., anxiety, depression, cognitive capacity) may emerge under stressful situations. Future studies with larger samples would need to be designed explicitly to test this hypothesis.

To our knowledge, there are no previous studies where diurnal cortisol indices have been used to predict subsequent perceived stress after a long-term stressful event in healthy young adults. Our data has tentative similarities with previous cross-sectional studies, such as that of Ruiz-Robledillo et al. [76], who showed that high resilient coping was associated with low cortisol and better-perceived health and social support. Our moderation model showed high resilient coping (+1 SD) at low cortisol levels (−1 SD) also predicts lower perceived stress during confinement (−1 SD). Moreover, low AUCg (−1 SD) at low resilience levels predicts high perceived stress (+1 SD), while for CAR, at very low resilience levels (8.25 BRCS score and below), flatter CAR responses correlate with high perceived stress (+1 SD). This result draws attention to the study by O’Connor and colleagues [77], who reported that high perceived stress predicted flattened CAR profiles. We found that pre-pandemic AUCg, but not CAR, were related to depressive-like symptoms and anxiety due to the COVID-19 pandemic. Among the sparse relevant literature, Lemoult et al. [35] reported similar results in that young girls with high AUCg indices showed a higher susceptibility to onset of depression later in adolescence after experiencing negative life events, but this was not observed in girls with a high CAR index. In line with our results, other authors reported no direct relation between CAR and depressive symptoms [78], while certain studies report CAR to be predictive of depressive symptoms [79,80,81]. Our findings are in line with the diathesis-stress model of depression, postulating that prodromal vulnerability factors, such as the altered function of the HPA axis, interact with environmental stressors to increase the risk for depression [68,82]. Thus, it might be hypothesized that high diurnal cortisol levels occur in those individuals with a greater stress-related arousal state and more susceptible to the impact of stressors. Although it is not well understood how a higher AUCg can increase susceptibility to stress, in depressed patients, it has been proposed that elevated diurnal cortisol levels can alter functioning in brain areas that exert HPA-axis negative feedback loops [27,83] and play a crucial role in emotional processing hindering the ability to cope with future stressful events [84,85]. Hence, our results suggest that a high AUCg during pre-pandemic might be considered an early biomarker of inefficient HPA-axis negative feedback, which, in turn, can alter individuals’ reactivity to stressful events.

Overall, the finding that resilient coping can increase or decrease perceived stress at different basal diurnal cortisol conditions may be relevant to understand the often-reported contradictory results obtained across studies exploring relations between psychological and physiological stress, documenting a negative relation [86,87], no relation [88] or a positive relation [89,90,91].

Our second objective was to examine the relationship between cortisol, cognition, and stress perception with the specific prediction (Hypothesis 2) that changes in cognitive capacity would mediate the relationship between pre-pandemic cortisol and the changes in perceived stress (Figure 1b). We used the dynamic values obtained via subtracting the during-confinement scores from the pre-pandemic scores for a concise representation of the neuropsychological effects of COVID-19. We expected a predictive relationship between cortisol and cognition owing to the study by Moriarty et al. [51]. The authors showed CAR’s inverted-U association with spatial working memory, the same task explored in the current study using the Corsi-block tapping test. Interestingly, not only working memory performance, but also the ability of perspective-taking relies on the prefrontal cortex [43,92]. Additionally, the prefrontal cortex is one of the brain structures that exerts a negative feedback on the HPA axis activity [93], while CAR occurs during post-awakening reversal of sleep inertia and reactivation of the prefrontal cortex activity [94]. The mediation model presents results in-line with the above, given that CAR and AUCg indices are related to change in spatial working memory and perspective-taking (cognitive empathy). The model further shows that the increase in working memory and perspective-taking, in turn, correlates with a decrease in the worsening of perceived self-efficacy (keeping in mind perceived self-efficacy was the driving factor behind total perceived self-efficacy being predicted in the moderation models). 

Previously, investigators have demonstrated that both genetic and cognitive factors could be implicated in the response to stress [95]. In addition, it has been reported that after adverse circumstances/events, those subjects with higher cognitive abilities showed more positive results, such as better academic performance and better social acceptance (implicating empathy) and friendship [46,96]. Otto et al. [97] showed that perceived improvement in problem-solving was associated with lower perceived stress. Improved cognition may allow for more cognitive capacity to process and use the novel circumstances to more efficiently manage them [98], and this could, in turn, influence change in perceived self-efficacy. In our view, this effect of higher cognition also applies to improvement in perspective-taking correlating with an attenuated decrease in perceived self-efficacy as seen in the current study. Although there are few studies on the matter and results are inconclusive, Gambin and Sharp [99] found an inverse relationship between cognitive empathy and social/separation anxiety in inpatient adolescents. Apart from the indirect effect of cortisol indices (both AUCg and CAR) on perceived self-efficacy, AUCg also directly affects perceived self-efficacy albeit in the opposite direction. AUCg was directly related to a worsening of perceived self-efficacy while indirectly, through cognitive capacities, it related to an improvement in self-efficacy after COVID-19 quarantine. Such suppression by the two effects on one another, leading to an overall total effect being non-significant, is a known cause of missing relevant relations [100] and may partly explain why the relationship between cortisol and perceived stress has been elusive.

We also observed that compared to before the pandemic, strict long-term confinement during the COVID-19 pandemic led to a significant increase in perceived stress, visuospatial working memory, and trait-like perspective-taking in young adults. An increase in perceived stress was not surprising given not only the fear of infection but also the uncertainty caused by the preventive measures (e.g., pre-emptive quarantines) and the ensuing change in routine [101,102]. This increase in uncertainty is apparent when looking at the two constructs that the perceived stress scale evaluates; perceived helplessness and perceived self-efficacy. Our results indicated that the increase in total perceived stress was driven primarily by an increase in perceived helplessness. This is congruous with the condition the general public has found itself in; obliged to stay inside their homes to avoid an invisible threat the subjects, as individuals, have little apparent control over.

Concerning the significant increase in the performance of the Corsi block-tapping test, a measure of visuospatial working memory span, we consider this result to be an observation of the bidirectional effect of stress on cognitive abilities [103,104,105]. Thus, although the pandemic has caused an increase in stress, we may speculate that the intensity of this stress has not been high enough to be detrimental to our subjects’ short-term working memory. One argument against this conclusion could be that there is a learning effect caused by the repetition of the cognitive task. However, on top of the 6-month gap between the two tests, previous research has noted the absence of any learning-effect when Corsi is repeated from traditional versions to e-Corsi [106,107]. Nor are there any differences between Corsi administered face-to-face in laboratory settings or via the use of e-Corsi [108]. If anything, Claessen et al. [109] observed results where traditional Corsi-forward reproduction resulted in higher accuracy compared to e-Corsi. On the other hand, short-term working memory to maintain objects in a spatial series demands active spatial attention [110,111]. This implies a direct role of attention scope and control in Corsi memory span [56,112]. Therefore, it may be argued that improved performance in the Corsi test is not a reflection of better working memory span ***per se***, but it is an effect of an increase in working memory capacity. However, the absence of differences between pre-pandemic and during-confinement change-location task scores discards the possibility that the pandemic and the associated confinement had changed focal attention capacity specifically. This result is in line with previous research showing that changes in positive or negative emotion/mood had no impact on spatial attention [113].

Similar to working memory, we observed an improvement in perspective-taking but no change in empathic concern, as measured by the IRI, a self-report instrument measuring dispositional empathy. Perspective-taking and mentalizing are empathy processes that require more complex cognitive systems [114]. Thus, perspective-taking has been shown to be dependent on the prefrontal cortex [43,115]. Thereby, our observation of an increase in perspective-taking is in line with the improvement in cognitive systems as seen with working memory, a cognitive process also governed by the prefrontal cortex (for rev. see [92]). Additionally, the absence of an increase in empathic concern, a process not strongly related to the prefrontal cortex and cognitive systems [116], is also worth noting.

Although our study provides new information not only about the relationships between cognitive capacities, psychological stress, and specifically, the basal HPA cortisol indices but also regarding the impact of the COVID-19 epidemic, some limitations should be considered when interpreting the results of this study. Because of the circumstances, the sample size of the study was constrained, and owing to the declared national emergency, timely distribution of saliva collection tubes at the home confinement stage could not be achieved. Similarly, the sample size determines that additional important factors (e.g., residence type, economic concerns, exposure to SARS CoV-2 coronavirus) cannot be controlled for in our models. Additionally, testing during home-confinement had to be done with subjects at their residence and without supervision, a fact that may have biased those results. Given how widespread COVID-19 has been, it was not possible to have a control group, which did not pass through confinement. This should be kept in mind when interpreting the differences observed between the pre-pandemic and during confinement phases of the study. While seasonal changes tend to impact depression-like symptoms in the direction contrary to our results [117], there may be other factors involved in these changes, apart from the situation caused by the COVID-19 pandemic. Importantly, although we have some specific hypotheses, several analyses were performed, and we carried out a number of exploratory analyses (e.g., analyses with subscales of the PSS). Given the number of analyses and the concerns regarding type I error, more research is needed to confirm the results of this study. Finally, it is possible that performing the neuropsychological evaluation in the earlier weeks of the confinement, instead of at the end, may have yielded different results. As a note of caution, we suggest these results be indicative, given how few studies there are about natural stressors of this kind and the inherent complexity as compared to laboratory settings.

## 5. Conclusions

This study presents new data concerning how people confront long-term stressors (such as the COVID-19 pandemic confinement) that can help advance understanding of how the impact of such crises varies according to individual HPA-axis set-up. The results support the potential relevance of diurnal cortisol indices in a clinical setting as biomarkers helping predict individual vulnerability to the onset of stress-related disorders. Our findings argue for attention to coping ability, cognitive function, and overall contextual landscape of the stimuli under study for a more complete interpretation of the dynamic between physiological and psychological stress.

## Figures and Tables

**Table 1:** Characteristics of the study sample for the cohort that participated in both phases of the study. Scores at pre-pandemic (diurnal cortisol secretion (AUCg) and cortisol awakening response (CAR) and during-confinement (anxiety, depression, resilient coping) sessions of the study.

	Mean (SD)
Gender	80% female
Age	21.09 (6.42)
Ethnicity	93.6% Caucasian
AUCg	3.880 (1.492)
CAR	0.126 (0.115)
BRCS	13.911 (3.636)
DASS (Anxiety)	8.356 (6.079)
DASS (Depression)	9.422 (5.864)

Note. SD = Standard Deviation; AUCg (μg/dL) = Total diurnal cortisol release; CAR (ug/dL) = Cortisol Awakening Response; BRCS = Brief Resilient Coping Score; DASS = Depression, Anxiety and Stress Scale.

**Table 2:** Pre-pandemic and during-confinement scores for the cohort that participated in both phases of the study.

	Pre-Pandemic	During-Confinement
IRI: Perspective-Taking	18.93(3.66)	20.56(3.53)
IRI: Empathic Concern	22.67(3.30)	22.38(4.02)
PSS: Helplessness	17.51(5.21)	20.43 (5.30)
PSS: Self-Efficacy	12.95(5.67)	14.12(5.63)
PSS: Total	30.46(9.45)	30.46(9.70)
Corsi-Forward Score	8.88 (1.45)	9.66(1.45)
Corsi-Backward Score	7.77(1.54)	8.00(1.86)
Corsi-Total Score	16.66(2.52)	17.71(2.61)
Change-Location Score	3.17(0.35)	3.26(0.38)

Note. Mean and Standard Deviation (SD) presented; AUCg (ug/dL) = Total diurnal cortisol release; CAR (ug/dL) = Cortisol Awakening Response; IRI = Interpersonal Reactivity Index; PSS = Perceived Stress Scale.

## Data Availability

The data presented in this study are available on request from the corresponding authors.

## Supplementary Materials

The following are available online at https://www.mdpi.com/2076-3425/11/3/348/s1, Figures S1–S3: Conditional effects simple slopes for AUCg/CAR and PSS/DASS, Figure S4: Mediation models while controlling for sex and age, Figure S5: Conditional effects with the unstandardized scores for AUCg and PSS, Table S1: Moderation tables for AUCg/CAR and PSS/DASS, Table S2: Non-participant and participant cohort means, Table S3: Moderation models with age and sex as covariables.
