# Supplementary material for: Differential Susceptibility to the Impact of the COVID-19 Pandemic on Working Memory, Empathy, and Perceived Stress: The Role of Cortisol and Resilience
Source: Brain Sci. 2021 Mar 9;11(3):348. doi: 10.3390/brainsci11030348 (PMC7998983; doi:10.3390/brainsci11030348)

Table S1: Moderation models.

predictors	Model 1 - Depression (DASS)				Model 2 - Anxiety (DASS)			
	β	t	LLCI: ULCI	Δr^2	β	t	LLCI: ULCI	Δr^2
AUCg	-.306(.140)	-2.183	-.591: -.021		-.157(.153)	-1.024	-.467: .155	
BRCS	-.038(.157)	-.244	-.357: -.280		-.014(.172)	-.081	-.335: .363	
AUCg*BRCS	.537(.178)	3.012	.175: .900	.192	.535(.196)	2.741	.138: .932	.173

predictors	Model 3 - Depression (DASS)				Model 4 - Anxiety (DASS)			
	β	t	LLCI: ULCI	Δr^2	β	t	LLCI: ULCI	Δr^2
CAR	-.162(.151)	-1.075	-.468: .144		-.084(.160)	.525	-.240: .408	
BRCS	-.250(.156)	-1.604	-.566: .066		-.198(.165)	-1.201	-.532: .136	
CAR*BRCS	.220(.200)	1.098	-.187: .626	.030	.104(.212)	.493	-.325: .534	.007

predictors	Model 5 - PSS ([lack of] Self-Efficacy)				Model 6 - PSS (Helplessness)			
	β	T	LLCI: ULCI	Δr^2	β	T	LLCI: ULCI	Δr^2
AUCg	-.253(.127)	-1.984	-.511: .006		-.190(.151)	-1.262	-.498: .117	
BRCS	-.254(.142)	-1.780	-.543: .035		-.022(.170)	.129	-.322: .366	
AUCg*BRCS	.668(.162)	4.121	.339: .997	.249	.513(.193)	2.663	.122: .905	.166

predictors	Model 7 - PSS ([lack of] Self-Efficacy)				Model 8 - PSS (Helplessness)			
	β	t	LLCI: ULCI	Δr^2	β	t	LLCI: ULCI	Δr^2
CAR	-.148(.131)	-1.128	-.414: .119		-.042(.157)	-.270	-.360: .276	
BRCS	-.493(.136)	-3.634	-.768: -.218		-.170(.162)	-1.052	-.497: .158	

AUCg*BRCS	.569(.174)	3.266	.215: .923	.174	.180(.208)	.864	-.243: .602	.020

Note. β =Standardized effect size; SE=Standard Error; DASS=Depression, Anxiety and Stress Scale; LLCI=Lower Limit of Confidence Intervals 95%; ULCI=Upper Limit of Confidence Intervals 95%; AUCg=Cortisol index for area under curve from ground; BRCS=Brief Resilient Coping Score; CAR=Cortisol Awakening response. Models 1, 2, 5, 6, 7: conditional effects (for 16th, 50th and 84th percentile) graphs below

Figure S1: Conditional effects simple slopes with absolute scores for model 1 (figure (a)) and 2 (figure (b)):

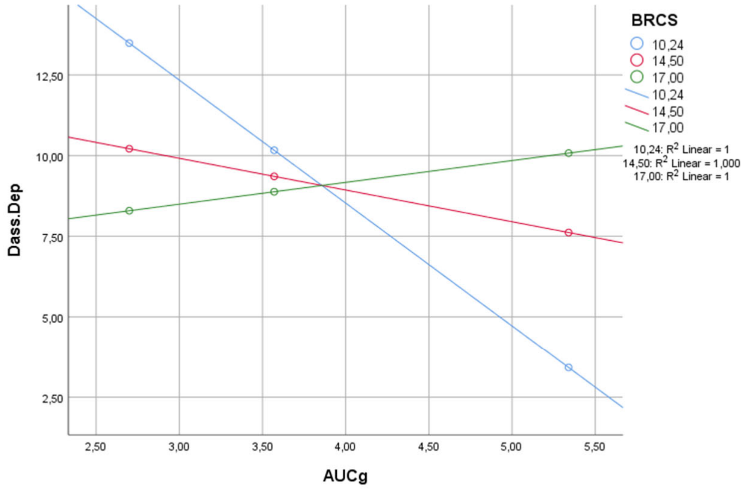

(a)

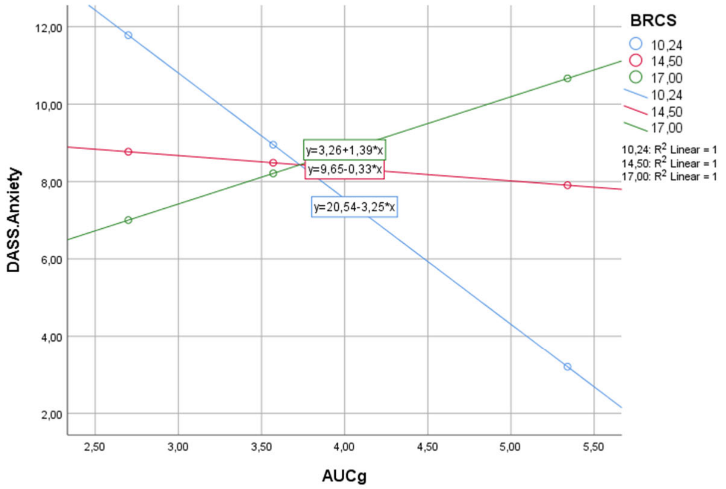

(b)

Note. AUCg=Total cortisol index for the day; BRCS=Brief Resilient Coping Score; DASS=Depression, Anxiety and Stress Scale

Figure S2: Conditional effects simple slopes with absolute scores for model 3 (figure (c)) and 4 (figure (d)):

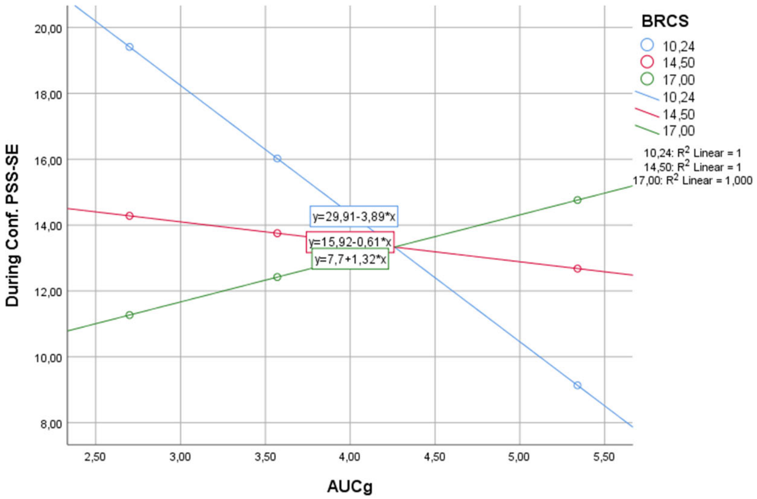

(c)

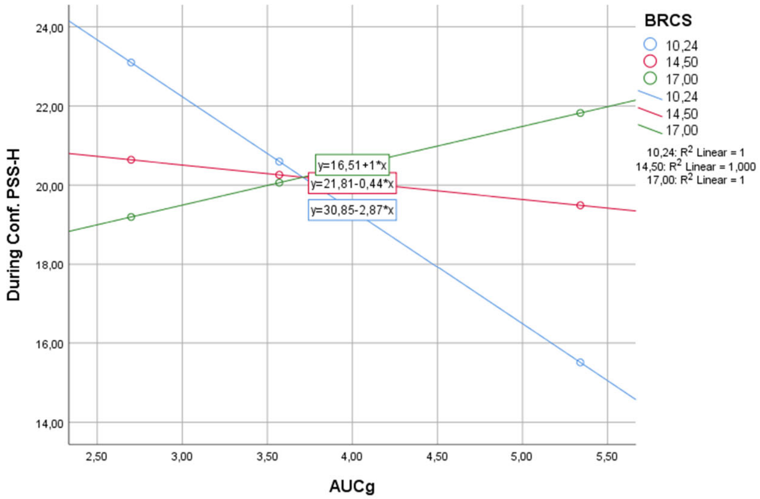

(d)

Note. PSS-SE=Perceived Stress Scale-Self-Efficacy; PSS-H=Perceived Stress Scale-Helplessness; AUCg=Total cortisol index for the day.

Figure S3: Conditional effects simple slopes with absolute scores for model 7 (figure (e)):

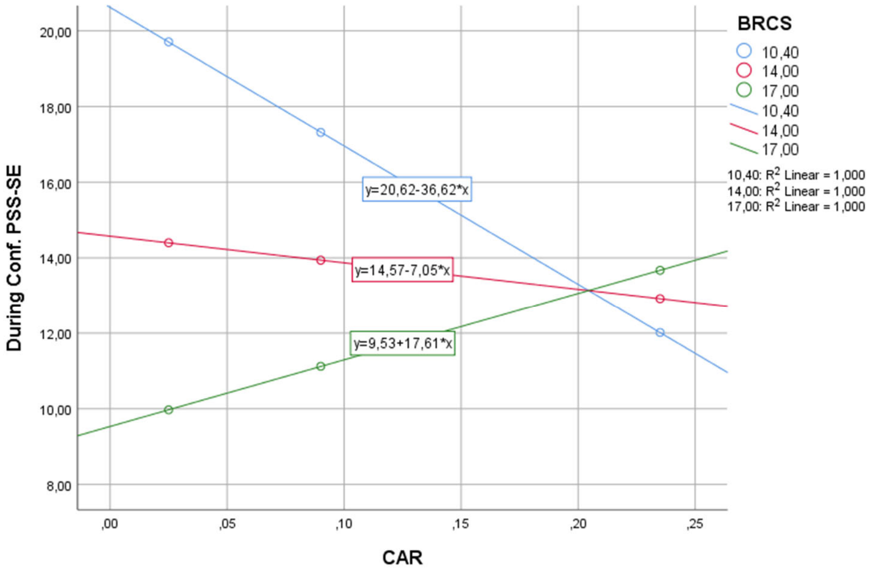

(e)

Note. PSS-SE=Perceived Stress Scale-Self-Efficacy; BRCS=Brief Resilient Coping Score; CAR=Cortisol Awakening Response.

Table S2. Scores at pre-pandemic stage for the subject pool which abstained from participating in the during confinement phase and scores for the 'during confinement' cohort.

	Non-participant cohort	During confinement cohort
**AUCg**	3.61(1.02)	3.89(1.49)
**CAR**	0.10(0.09)	0.12(0.11)
**Age**	20.12(2.50)	21.09(6.42)
**IRI: Perspective Taking**	20.09(4.22)	18.93(3.66)
**IRI: Empathic Concern**	21.74(4.33)	22.67(3.30)
**PSS: Helplessness**	16.17(5.23)	17.51(5.21)
**PSS: Self-Efficacy**	11.25(4.09)	12.95(5.67)
**PSS: Total**	27.42(8.31)	30.46(9.45)
**Corsi-Forward Score**	8.66(1.56)	8.88 (1.45)
**Corsi-Backward Score**	8.00(1.27)	7.77(1.54)
**Corsi-Total Score**	16.66(2.27)	16.66(2.52)
**Change-Location Score**	3.08(0.39)	3.17(0.35)

Note. Mean and Standard Deviation (SD) presented; AUCg (ug/dL)=Total diurnal cortisol release; CAR (ug/dL)=Cortisol Awakening Response; IRI=Interpersonal Reactivity Index; PSS=Perceived Stress Scale.

Table S3: Moderation models with Age and Sex as covariables.

predictors	Model A – AUCg to Perceived stress				Model B – AUCg to DASS-Depression			
	b(SE)	t	LLCI: ULCI	Δr^2	b(SE)	t	LLCI: ULCI	Δr^2
AUCg	-21.036(5.612)	-3.747	-32.469: -9.602		-11.060(3.506)	-3.154	-18.203: -3.918	
BRCS	-5.435(1.216)	-4.467	-7.913: -2.957		-2.595(.760)	-3.414	-4.143: -1.047	
AUCg*BRCS	1.374(.362)	3.792	.656: 2.112	.262	.689(.226)	3.041	.227: 1.150	.201
Sex	-1.063(3.540)	-.300	-8.273: 6.147		-.213(2.211)	-.096	-4.718: 4.294	
Age	-.165(.299)	-.551	-.774: .444		-.186(.187)	-.995	-.566: .195	

predictors	Model C – AUCg to DASS-Anxiety							
	b(SE)	t	LLCI: ULCI	Δr^2				
AUCg	-11.1410(3.895)	-2.859	-19.076: -3.205					
BRCS	-2.6139(.8445)	-3.095	-4.334: -.893					
AUCg*BRCS	.7354(.251)	2.923	.223: 1.247	.195				
Sex	-1.098(2.456)	-.447	-6.102: 3.906					
Age	-.321(.207)	-1.548	-.744: .101					

predictors	Model D – CAR to Perceived stress							
	b(SE)	t	LLCI: ULCI	Δr^2				
CAR	-169.988(78.240)	-2.173	-329.172: -10,804					
BRCS	-2.198(.668)	-3.291	-3.556: -.8389					
CAR*BRCS	11.493(5.205)	2.208	.904: 22.089	.108				
Sex	-2.008(4.091)	-.491	-10.332: 6.316					

Age	-.109(.334)	-.329	-.785: .587					

Note. b=Unstandardized effect size; SE=Standard Error; DASS=Depression, Anxiety and Stress Scale; LLCI=Lower Limit of Confidence Intervals 95%; ULCI=Upper Limit of Confidence Intervals 95%; AUCg=Cortisol index for area under curve from ground; BRCS=Brief Resilient Coping Score; CAR=Cortisol Awakening response.

Figure S4: Mediation models while controlling for sex and age: (a) AUCg cortisol predicting change in perceived self-efficacy; (b) CAR predicting change in perceived self-efficacy. PT=Perspective Taking; PSS-SE=Perceived stress scale Self-Efficacy; AUCg=Total diurnal cortisol release; CAR=Cortisol Awakening Response; ns=non-significant. Standardized effect sizes in red. Accompanying tables for figures (a) and (b) present data about standardized total and individual indirect effects of cortisol indices (AUCg and CAR) on change in perceived self-efficacy.

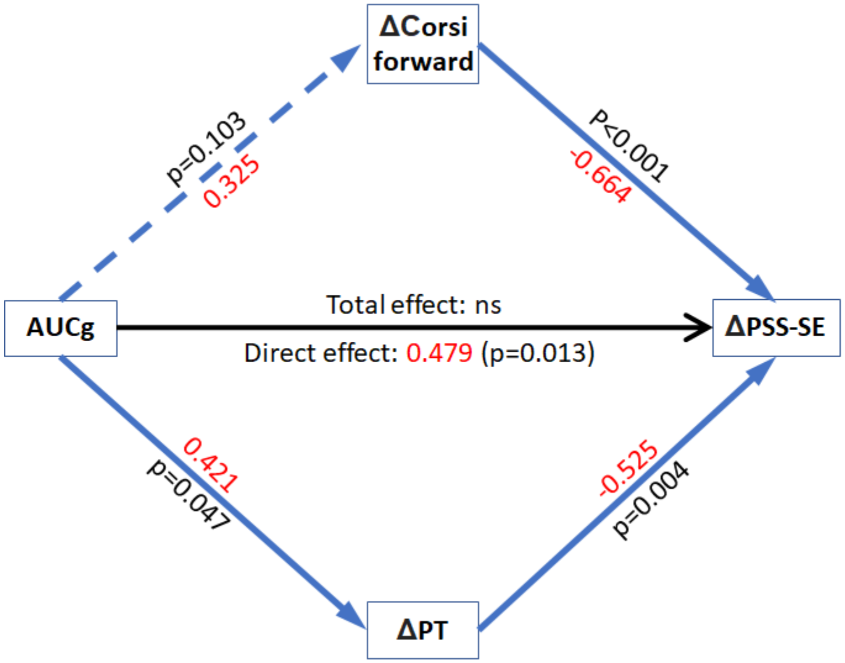

(a)

Indirect effect(s) of AUCg on ΔPSS-SE:

**Via**	Effect	Std. Error	LLCI: ULCI
Total	-.437	.157	-.784: -.151
ΔPT	-.221	.117	-.476: -.036

ΔCorsi-forward	-.216	.114	-.540: .044

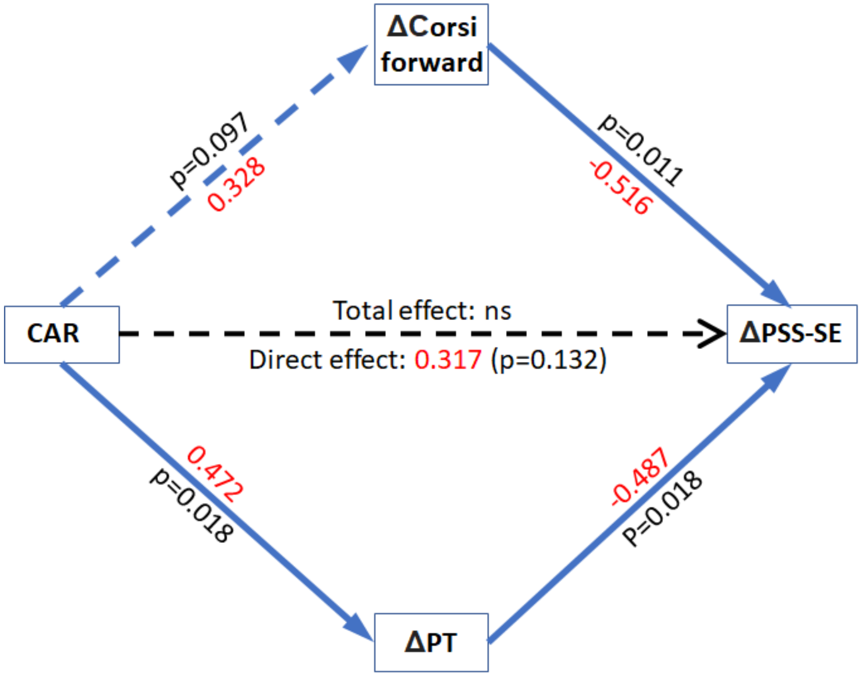

(b)

Indirect effect(s) of CAR on ΔPSS-SE:

**Via**	Effect	Std. Error	LLCI: ULCI
Total	-.399	.143	-.656: -.100
ΔPT	-.230	.113	-.455: -.017
ΔCorsi-forward	-.169	.100	-.384: .016

Figure S5: Simple slopes (conditional effects) representing the association, in raw scores, between Resilient Coping and pre-pandemic daytime cortisol AUCg predicting confinement total perceived stress. BRCS=Brief Resilient Coping Score; AUCg=Total diurnal cortisol release; PSS=Perceived Stress Score.

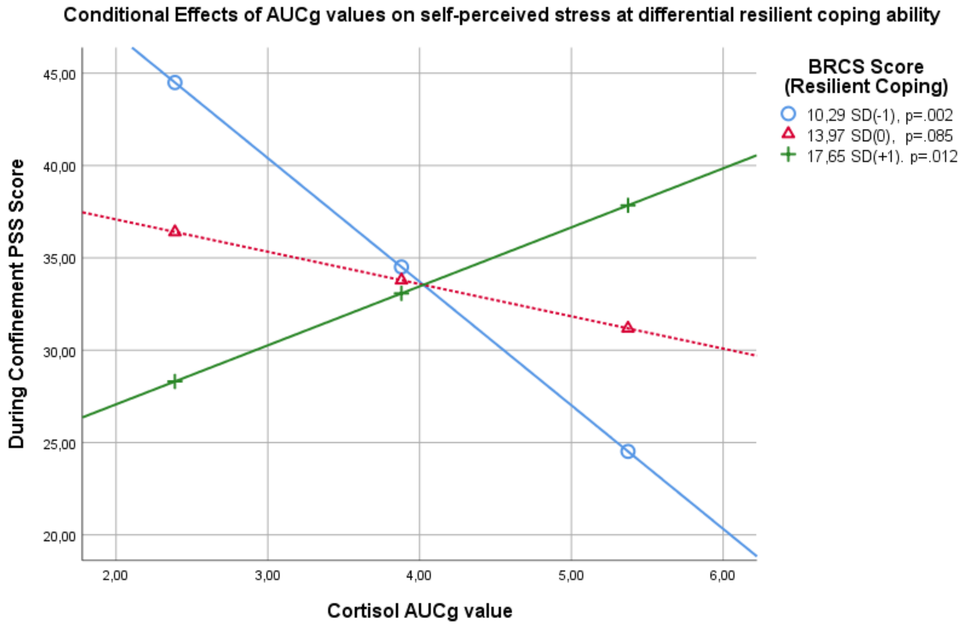

Supplement: Supplementary file 1 [file brainsci-11-00348-s001.pdf]
